# Disrupted Brain Structural Network Connection in *de novo* Parkinson's Disease With Rapid Eye Movement Sleep Behavior Disorder

**DOI:** 10.3389/fnhum.2022.902614

**Published:** 2022-07-19

**Authors:** Amei Chen, Yuting Li, Zhaoxiu Wang, Junxiang Huang, Xiuhang Ruan, Xiaofang Cheng, Xiaofei Huang, Dan Liang, Dandan Chen, Xinhua Wei

**Affiliations:** ^1^Department of Radiology, The First Affiliated Hospital of Jinan University, Guangzhou, China; ^2^Department of Radiology, Guangzhou First People's Hospital, School of Medicine, South China University of Technology, Guangzhou, China; ^3^Department of Anesthesiology, Guangzhou Women and Children's Medical Center, Guangzhou, China; ^4^Department of Radiology, Affiliated Brain Hospital of Guangzhou Medical University, Guangzhou, China

**Keywords:** rapid eye movement sleep behavior disorder (RBD), Parkinson's disease, diffusion tensor imaging, graph theory, topological property, structural brain network

## Abstract

**Objective:**

To explore alterations in white matter network topology in de novo Parkinson's disease (PD) patients with rapid eye movement sleep behavior disorder (RBD).

**Materials and Methods:**

This study included 171 de novo PD patients and 73 healthy controls (HC) recruited from the Parkinson's Progression Markers Initiative (PPMI) database. The patients were divided into two groups, PD with probable RBD (PD-pRBD, *n* = 74) and PD without probable RBD (PD-npRBD, *N* = 97), according to the RBD screening questionnaire (RBDSQ). Individual structural network of brain was constructed based on deterministic fiber tracking and analyses were performed using graph theory. Differences in global and nodal topological properties were analyzed among the three groups. After that, post hoc analyses were performed to explore further differences. Finally, correlations between significant different properties and RBDSQ scores were analyzed in PD-pRBD group.

**Results:**

All three groups presented small-world organization. PD-pRBD patients exhibited diminished global efficiency and increased shortest path length compared with PD-npRBD patients and HCs. In nodal property analyses, compared with HCs, the brain regions of the PD-pRBD group with changed nodal efficiency (Ne) were widely distributed mainly in neocortical and paralimbic regions. While compared with PD-npRBD group, only increased Ne in right insula, left middle frontal gyrus, and decreased Ne in left temporal pole were discovered. In addition, significant correlations between Ne in related brain regions and RDBSQ scores were detected in PD-pRBD patients.

**Conclusions:**

PD-pRBD patients showed disrupted topological organization of white matter in the whole brain. The altered Ne of right insula, left temporal pole and left middle frontal gyrus may play a key role in the pathogenesis of PD-RBD.

## Introduction

Rapid eye movement sleep (REM) behavior disorder (RBD), which is characterized by dream-related motor behavior and accompanied by phasic tension electromyography during REM sleep, is one of the most common non-motor symptoms of Parkinson's disease (PD) (Gagnon et al., 2002; Sudarsky and Friedman, 2006). RBD is considered a prodrome of PD, with an incidence of 25% in new PD patients and more than 50% in the entire PD population (Marion et al., 2008; Destrieux et al., 2010; Kalia and Lang, 2015). In addition, evidences indicate that RBD in PD patients is associated with greater motor and non-motor symptoms, such as smell disorders, constipation, visual hallucinations, cognitive impairment, depression, and impulse control disorders (Agosta et al., 2015; Arnaldi et al., 2016). However, the potential neurological mechanisms underlying RBD in such patients are yet to be elucidated.

A handful of magnetic resonance imaging studies have explored the probable pathogenesis of PD with RBD. A longitudinal study (Yoon and Monchi, 2021) compared the changes in cortical thickness in PD patients with or without RBD. In comparison to the PD without probable RBD (PD-npRBD) subset, in the PD with probable RBD (PD-pRBD) group, the bilateral inferior temporal cortex was thinner at baseline and longitudinally, the thinning rate of the left insula was significantly increased. Some earlier PET and fMRI studies have described relative activation in limbic and paralimbic structures during REM sleep compared to that in nREM sleep and wakefulness (Braun et al., 1998; Nofzinger et al., 2002). Although these neuroimaging studies suggested that changes in cortical thickness or functional activity in paralimbic regions contributed to the appearance of RBD in PD, topological changes in the connectomes of the whole brain structure of PD-pRBD remain unclear.

In contrast to approaches that analyze localized brain changes, recent studies have viewed the human brain as a network model, as the structural and functional systems of the brain have the characteristics of complex networks. Graph theory is considered a good way to analyze such network. According to graph theory, network consists of nodes linked by edges, which can be quantitatively described through multiple measurements (Sporns et al., 2005; Bullmore and Sporns, 2009). In recent years, the white matter(WM) structural network connectome constructed by diffusion tensor imaging(DTI) has increasingly been analyzed with graph theory approaches, especially in PD patients, to determine alterations in the topological properties of brain structures (Pereira et al., 2015; Li C. et al., 2017; Li D. et al., 2017). Several neuroimaging studies using DTI have shown that compared with HCs, PD patients have lower whole-brain clustering coefficients and lower global efficiency (Nigro et al., 2016; Abbasi et al., 2018). However, it remains unclear whether PD-pRBD and PD-npRBD patients differ in WM connectivity.

Therefore, this study aimed to explore alterations in the WM structural network connectome in PD-pRBD patients and assess whether RBDSQ scores correlate with structural topological network changes, which might provide deeper insight into the neuropathological mechanisms underlying PD-RBD.

## Methods

### Participants and Clinical Evaluation

All MRI and clinical data were extracted from the Parkinson's Progression Markers Initiative (PPMI) database (www.ppmi-info.org/data), a multicenter longitudinal database of patients with Parkinson's disease (Parkinson Progression Marker Initiative., 2011). Each PPMI site was approved by its own ethical committee, and all PPMI subjects provided written informed consent prior to participating in the program. The analysis in this study was carried out according to the approved PPMI guidelines.

The PD cases included in the database were newly diagnosed and treatment-naïve; moreover, we selected only data from the baseline visits. Given that polysomnography is not currently available in the database, the presence of RBD in PD individuals was determined with the RBD screening questionnaire (RBDSQ). The RBDSQ has a sensitivity of 0.96 and specificity of 0.85 for the diagnosis of RBD when a cut-off score of 5 is used (Stiasny-Kolster et al., 2007). In the present study, PD patients with RBDSQ scores ≥ 5 were defined as having PD-pRBD, and those with RBDSQ scores <5 were defined as having PD-npRBD (Kamps et al., 2019). HCs with RBDSQ scores ≥ 5 (*n* = 1) were excluded. Patients with cognitive impairments (Montreal Cognitive Assessment (MoCA) score <23) (*n* = 4) were also excluded (Nasreddine et al., 2005). Finally, 74 PD-pRBD patients, 97 PD-npRBD patients, and 73 HCs were enrolled.

The neuropsychological performance of each participant was assessed with multiple cognitive and motor tests, including the Geriatric Depression Scale (GDS), Hopkins Verbal Learning Test (HVLT), Montreal Cognitive Assessment (MoCA), Scales for Outcomes in Parkinson's Disease-Autonomic (SCOPA-AUT), University of Pennsylvania Smell Identification Test (UPSIT) and Movement Disorder Society Unified Parkinson's Disease Rating Scale Part III (MDS-UPDRS-III).

### Image Acquisition

Non-contrast enhanced 3D volumetric T1-weighted MRI and DTI scans of the 244 total subjects were acquired on 3T Siemens MRI scanners (Erlangen, Germany) using an MP-RAGE sequence at different centers. Acquisition parameters and detailed protocols are available on the PPMI website. The following indexes were included in the protocol: (1) DTI: 72 axial slices, echo time (TE) = 88 ms, repetition time (TR) = 500–9,000 ms, voxel size: 2.0 × 2.0 × 2.0 mm^3^, acquisition matrix = 1,044 × 1,044; one diffusion-unweighted (b0) image and 64 diffusion-sensitive gradient directions at b = 1,000s/mm^2^ images; (2) 3D-T1WI: TE = 2.98 ms, TR = 2,300 ms, voxel size: 1.0 × 1.0 × 1.0 mm^3^, acquisition matrix = 240 × 256. Staff at each center were trained to ensure that the data were collected in a standardized manner. In subsequent data analysis, subjects with missing data were excluded.

### DTI Preprocessing and Tractography

PANDA software (Cui et al., 2013) was used to preprocess DTI images, which mainly including the following: First, the non-brain tissue was stripped off. Second, the eddy-current effect and mild head motion were corrected. Then, the diffusion tensor (DT) matrix was calculated based on the voxel. Finally, the fiber assignment by continuous tracking (FACT) algorithm was applied to conduct deterministic tractography. If the curvature angle was greater than 45° or the voxel FA < 0.2, the trace was terminated.

### Network Construction

The T1WI images of all individual subjects were first mapped to the corresponding B = 0 s/mm^2^ images to obtain jointly calibrated T1 images in the DTI space. Then, the transformed T1 images were non-linearly transformed into ICBM152 T1 template in Montreal Neuroscience Institute (MNI) space. According to the automated anatomical labeling (AAL) atlas of the MNI, the brain can be divided into ninety regions, which serve as nodes of the brain structural network (Supplementary Table 1). A 90×90 weighted matrix was obtained by taking the number of fibers (FN) in each pair of brain regions as the threshold value T. We set T ≥3 as a threshold indicating the existence of fiber connections between brain regions, which was defined as 1; otherwise, this value was defined as (Shu et al., 2009). PANDA was used to establish a binary matrix of the whole-brain network.

### Graph Analysis of Network Topology

All network analyses were performed using the graph theory network analysis toolbox GRETNA (https://www.nitrc.org/projects/gretna), and the results were visualized by using the BrainNet Viewer toolbox (http://www.nitrc.org/projects/bnv).

### Global Properties of the Network

The global properties of the network include the global efficiency (Eg), local efficiency (Eloc), cluster coefficient (Cp), shortest path length (Lp), and “small-worldness” (σ) (Wang et al., 2020). Eg is an important parameter for estimating the transmission efficiency of the global network. Eloc describes the network interconnection between nodes. Cp represents the average clustering coefficient of all nodes and is mainly used to measure the level of network interconnectivity. Lp refers to the average length of the shortest path between any two nodes in the network; a smaller Lp value represents a higher efficiency of network information transmission. “Small-world” networks have higher clustering coefficients and smaller shortest path lengths, which ensures that information is the efficiently transmitted both globally and locally. The study generated 1,000 random networks and compared them with real networks to explore subjects' “small-worldness.” γ and λ represent the ratios of Cp and Lp in real networks to those in random networks, respectively. σ is defined as the ratio of γ to λ. When γ > 1 and λ ≈ 1, or σ > 1, the network was regarded as a ”small-world" network (Li C. et al., 2017; Li D. et al., 2017).

### Nodal Properties and Hubs

The nodal properties of a network include nodal efficiency (Ne), nodal shortest path length (Nlp), nodal cluster coefficient (Ncp), nodal degree centrality (Dc) and node betweenness (Bc). Ne refers to the efficiency of information transfer between a node (i) and all other nodes in the network. Nlp refers to the shortest path from one node to all other nodes. Ncp refers to the density of connections between the neighbors of a node. Dc is defined as the number of edges shared by node (i) and other nodes in the network. Bc refers to the fraction of the shortest paths through node (i) (Wang et al., 2020).

In the 90 included nodes, a number of specific nodes interact with many other brain regions; these were defined as hubs. Hubs play an important role in maintaining network stability. We defined nodes with a high Dc as hubs (the centrality of the hub node ≥ the average centrality + 1 SD) (Hosseini and Hoeft, 2012).

### Statistical Analysis

The demographic characteristics and clinical data were compared with IBM SPSS 22 software among the three groups. The distribution-based chi-square test was applied to compare qualitative data, and one-way ANOVAs or Kruskal–Wallis H tests were used for quantitative data. The significance level was *p* < 0.05.

Significance values were adjusted by the Bonferroni correction for multiple comparisons. After controlling for age, sex, and years of education, a one-way ANOVA was applied to compare differences in global and local attributes among the groups. For global parameters, *p* < 0.05 was considered significant; for nodal parameters, significance values were adjusted by the Bonferroni correction for multiple tests. Subsequently, post hoc analyses were performed to further explore differences between the two groups: two-sample t tests were applied for post hoc analyses of significant nodal properties and the significant values were adjusted by the Bonferroni correction. All graph theory parameters were analyzed using the GRETNA statistical model.

Finally, multiple linear regression analysis was applied to test the relationship between structural network parameters and RBDSQ scores in the PD-pRBD group.

## Results

### Demographic and Clinical Variables

A total of 244 participants were enrolled (73 HC, 97 PD-npRBD and 74 PD-pRBD patients). Demographic and clinical features are presented in Table 1. There were no significant differences among the three groups in age, sex or years of education (all *P* > 0.05). In addition, no significant differences were found among the three groups with respect to GDS, HVLT Immediate/Total Recall, HVLT Discrimination Recognition, HVLT Retention, MoCA, SCOPA-AUT, or UPSIT scores (*p* > 0.05). There was no significant difference in UPDRS-III scores between PD-pRBD and PD-npRBD patients (*p* > 0.05).

**Table 1:** Demographic and clinical characteristics of HC, PD-npRBD and PD-pRBD patients.

	**HC (*n* = 73)**	**PD-npRBD (*n* = 97)**	**PD-pRBD (*n* = 74)**	***P*** **values**
Age (years)	60.2 (10.1)	61.1 (9.3)	62.6 (9.7)	0.34 (F)
Sex (male%)	64.3%	63.9%	66.1%	0.74 (χ2)
Education (years)	16.06(3.45)	15.27(3.05)	15.36(2.89)	0.45 (F)
GDS	1.51 (2.99)	1.94 (2.29)	2.80 (2.81)	0.05 (H)
HVLT immediate/total recall	26.05 (4.72)	25.29 (5.34)	24.21 (5.18)	0.08 (H)
HVLT discrimination recognition	9.61 (3.67)	9.60 (3.19)	8.95 (3.42)	0.31 (H)
HVLT retention	0.91 (0.19)	0.88 (0.18)	0.80 (0.22)	0.28 (F)
MoCA	28.13 (1.15)	27.84 (1.94)	27.42 (2.26)	0.09 (F)
SCOPA-AUT	6.20 (3.04)	7.82 (4.01)	10.14 (5.78)	0.08 (H)
UPSIT	24.89 (10.06)	25.34 (8.62)	25.83 (9.48)	0.86 (F)
UPDRS-III	NA	28.09 (10.96)	26.08 (12.56)	0.57 (Z)

*HC, healthy control group; PD-npRBD Parkinson's disease with no probably REM Sleep Behavior Disorder; PD-pRBD Parkinson's disease with probably REM Sleep Behavior Disorder; GDS, Geriatric Depression Scale; HVLT, Hopkins Verbal Learning Test; MoCA, Montreal Cognitive Assessment; SCOPA-AUT, Scales for Outcomes in Parkinson's disease- Autonomic; UPSIT, University of Pennsylvania Smell ID Test; UPDRS-III, unified Parkinson's disease rating scale-third edition*.

*F, one-way ANOVA test; t, two-tailed t test; χ2, Chi square test; Z, Mann–Whitney U-test; H, Kruskal–Wallis test*.

Significant values were adjusted by the Bonferroni correction for multiple tests. ^*^The significance threshold was set at p < 0.05.

### Global Properties of the Network

**Figure 1:**
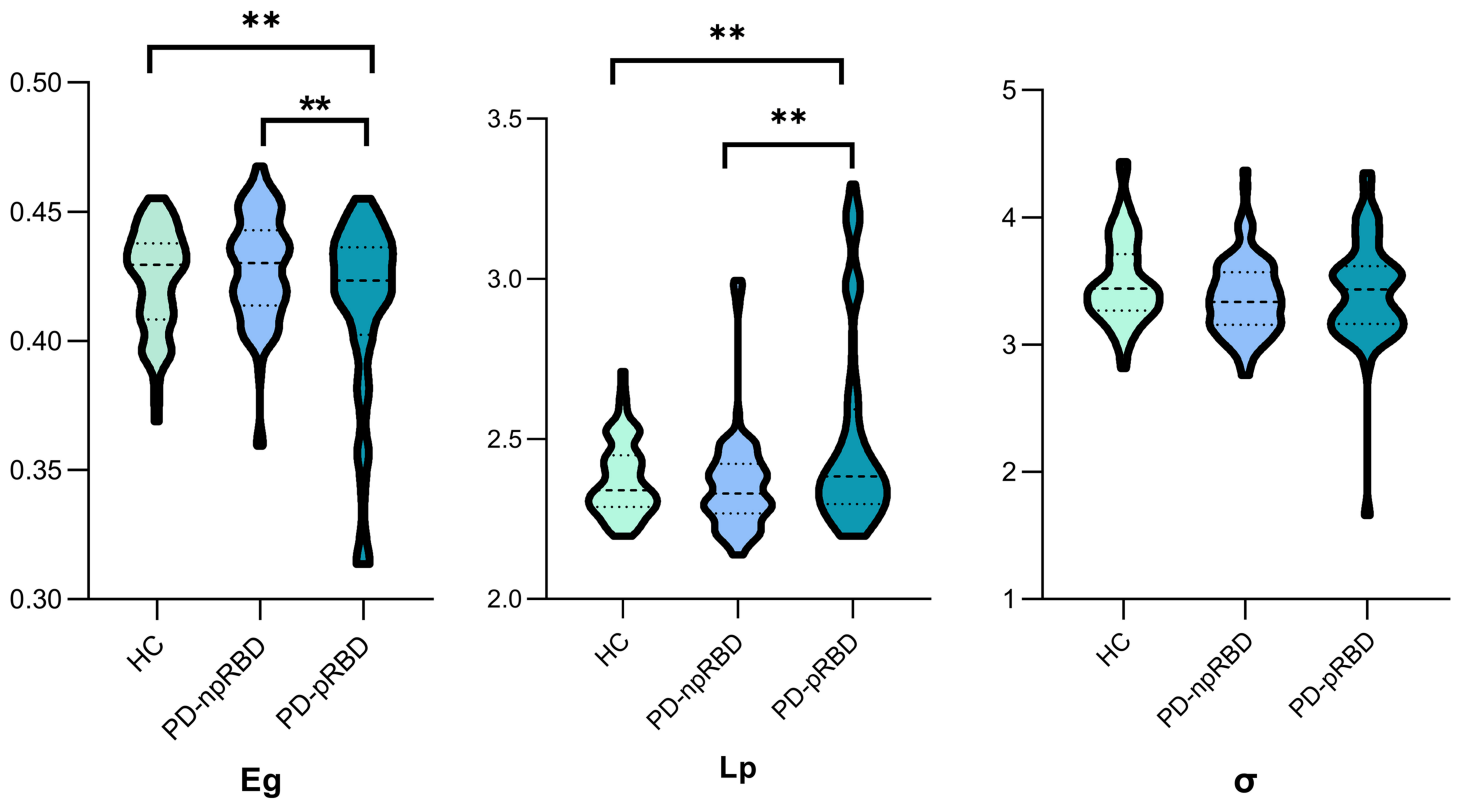
One-way ANCOVA of global parameters among the three groups. Significant differences of global parameters of the structural network were detected among the three groups, performed by one-way ANCOVA, adjusting for age, gender and education years. *p* < 0.05 was set significant. Afterwards, two-sample t-test was employed to explore further differences. **Post hoc tests were corrected by Bonferroni correction with a significant different *p* < 0.017 (0.05/3). Eg., global efficiency; Lp, shortest path length; σ, small-world index.

The brain WM structural networks of the HC, PD-npRBD and PD-pRBD groups all presented small-world organization (σ > 1), and there was no significant difference among the three groups. Significantly different Eg (F = 5.763; *P* = 0.002) and Lp (F = 5.739; *P* = 0.001) values were observed among the three groups with one-way ANOVA after adjusting for age, sex and education. Furthermore, PD-pRBD patients exhibited significantly decreased Eg (*t* = 3.480; *P* = 0.002) but increased Lp (*t* = −3.099; *P* = 0.003) values compared with PD-npRBD patients and HCs, after adjusting for age, sex, and education (Li D. et al., 2017). However, there were no significant differences in global topological properties detected between PD-npRBD patients and HCs (Figure 1).

### Nodal Properties and Hub Characteristics

#### Nodal Properties

**Figure 2:**
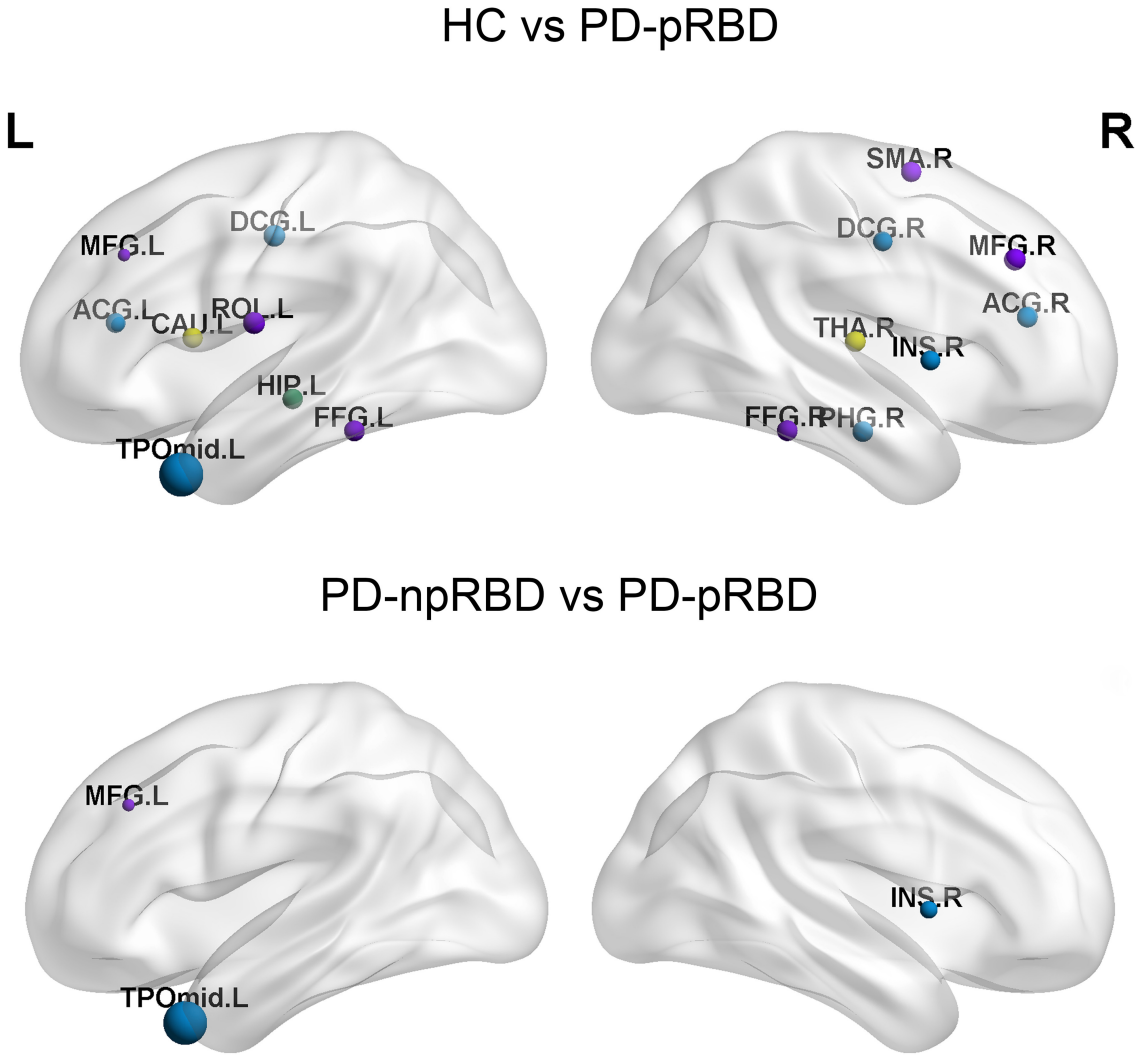
The distribution of brain regions with significant differences in nodal efficiency in HC, PD-npRBD and PD-pRBD groups. The size of the node reflects the value of the nodal efficiency. Different colors indicate a functional classification of brain regions: red, primary, purple, association, blue, paralimbic, green -limbic; yellow, subcortical. The abbreviations of the 90 brain regions are given in Supplementary Materials (Online Resource). L, left hemisphere; R, right hemisphere.

Nodal efficiencies of the WM networks of the HC, PD-npRBD, and PD-pRBD groups were analyzed. If significant differences among the three groups were observed (*P* < 0.05, Bonferroni corrected), then a post hoc test was performed. The results showed significantly increased Ne in the bilateral middle frontal gyrus, left rolandic operculum, right supplementary motor area, right insula, bilateral anterior cingulate and paracingulate gyri, bilateral median cingulate and paracingulate gyri, left hippocampus, right parahippocampal gyrus, bilateral fusiform gyrus, left caudate nucleus, and right thalamus (according to the AAL-90 atlas) in the PD-pRBD group compared to that in HCs, while the left temporal pole: middle temporal gyrus exhibited a significant reduction in Ne in the PD-pRBD group after adjusting for age, sex and education (P < 0.05, Bonferroni corrected). When compared with the PD-npRBD group, the PD-pRBD group had significantly increased Ne in the left middle frontal gyrus and right insula and decreased Ne in the left temporal pole: middle temporal gyrus, after adjusting for age, sex and education (P < 0.05, Bonferroni corrected) (Table 2; Figure 2). However, no significantly different nodes were detected between HCs and PD-npRBD patients. There were no significant differences in other nodal properties among the three groups.

**Table 2:** Between-group differences of nodal efficiency among HC, PD-npRBD, and PD-pRBD.

	**Regions**	**Functional classification**	**Anatomical classification**	**Difference value (*p* value)**
HC < pRBD	MFG.L	Association	Prefrontal	−3.93 (0.00)
HC < pRBD	MFG.R	Association	Prefrontal	−2.07 (0.04)
HC < pRBD	ROL.L	Association	Frontal	−2.01 (0.04)
HC < pRBD	SMA.R	Association	Frontal	−2.33 (0.02)
HC < pRBD	INS.R	Paralimbic	Subcortical	−2.46 (0.01)
HC < pRBD	ACG.L	Paralimbic	Prefrontal	−2.45 (0.02)
HC < pRBD	ACG.R	Paralimbic	Prefrontal	−2.22 (0.03)
HC < pRBD	DCG.L	Paralimbic	Frontal	−2.06 (0.04)
HC < pRBD	DCG.R	Paralimbic	Frontal	−2.51 (0.01)
HC < pRBD	HIP.L	Limbic	Temporal	−2.35 (0.02)
HC < pRBD	PHG.R	Paralimbic	Temporal	−2.28 (0.02)
HC < pRBD	FFG.L	Association	Temporal	−2.25 (0.03)
HC < pRBD	FFG.R	Association	Temporal	−2.28 (0.02)
HC < pRBD	CAU.L	Subcortical	Subcortical	−2.33 (0.22)
HC < pRBD	THA.R	Subcortical	Subcortical	−2.26 (0.03)
HC > pRBD	TPOmid.L	Paralimbic	Temporal	2.47 (0.01)
npRBD < pRBD	MFG.L	Association	Prefrontal	−3.22 (0.00)
npRBD < pRBD	INS.R	Paralimbic	Subcortical	−2.33 (0.02)
npRBD > pRBD	TPOmid.L	Paralimbic	Temporal	2.13 (0.03)

*The abbreviations of the 90 brain regions are given in supplementary materials (Online Resource)*.

#### Hub Characteristics

The following hubs were discovered in HCs: the left superior frontal gyrus, medial orbital, right calcarine fissure and surrounding cortex, left temporal pole: superior temporal gyrus, right temporal pole: middle temporal gyrus, left middle occipital gyrus, bilateral precuneus, and bilateral putamen. The following hubs were present in PD-npRBD patients: the bilateral temporal pole: superior temporal gyrus, left middle temporal gyrus, right superior occipital gyrus, left middle occipital gyrus, bilateral precuneus, and bilateral putamen. Additionally, the following hubs were discovered in PD-pRBD patients: the left insula, right superior frontal gyrus, orbital part, left precentral gyrus, right superior occipital gyrus, middle occipital gyrus, bilateral precuneus, and bilateral putamen (Table 3).

**Table 3:** Hubs characteristic of the HC, PD-npRBD, and PD-pRBD groups.

	**Hub regions**	**Class**	**Degree**
HC	ORBsupmed.L	Paralimbic	14
	CAL.R	Primary	19
	TPOsup.L	Paralimbic	14
	TPOmid.R	Paralimbic	16
	MOG.L	Association	15
	PCUN.L	Association	12
	PCUN.R	Association	18
	PUT.L	Subcortical	15
	PUT.R	Subcortical	12
PD-npRBD	**TPOsup.R**	**Paralimbic**	18
	**TPOsup.L**	**Paralimbic**	13
	**MTG.L**	**Association**	16
	SOG.R	Association	16
	MOG.L	Association	14
	PCUN.L	Association	11
	PCUN.R	Association	17
	PUT.L	Subcortical	14
	PUT.R	Subcortical	12
PD-pRBD	**INS.L**	**Paralimbic**	17
	**ORBsup.R**	**Paralimbic**	15
	**PreCG.L**	**Primary**	10
	SOG.R	Association	16
	MOG.L	Association	14
	PCUN.L	Association	12
	PCUN.R	Association	13
	PUT.L	Subcortical	11
	PUT.R	Subcortical	12

*The regions in bold represent different hub regions between the PD-npRBD and PD-pRBD groups. The abbreviations of the 90 brain regions are given in supplementary materials (Online Resource)*.

**Figure 3:**
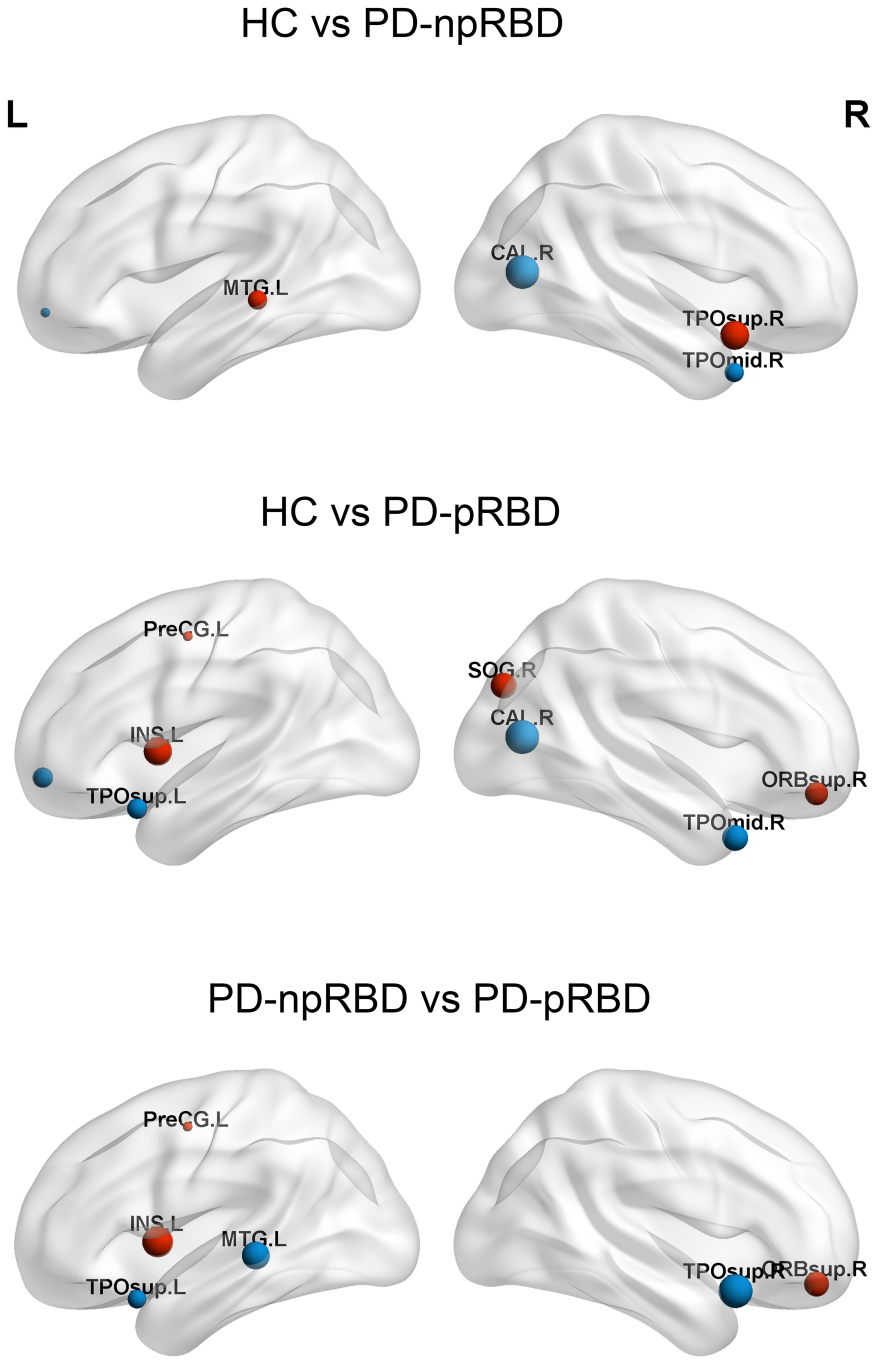
Differences of hubs in the HC, PD-npRBD, PD-pRBD. The size of the node reflects the value of the nodal degree. The blue dot represents the hub in the former group but not in the latter, and the red node is the opposite. The abbreviations of the 90 brain regions are given in Supplementary Materials (Online Resource). L, left hemisphere; R, right hemisphere.

Among the three groups, 5 hub regions (3 association cortices; 2 subcortical) were identical, including the left middle occipital gyrus, bilateral precuneus, and bilateral putamen. Three hub regions in PD-pRBD patients (the left insula, right superior frontal gyrus, orbital part, and left precentral gyrus) were not found in PD-npRBD patients, whereas 2 hubs in PD-npRBD patients (the bilateral temporal pole: superior temporal gyrus and left middle temporal gyrus) were not found in PD-pRBD patients (Figure 3).

As the *p* value of the GDS among the three groups is 0.05, we take GDS as covariate along with age, sex and years of education, and the results (including global properties and Nodal efficiency) were highly consistent with our main results (Supplementary Table 2; Supplementary Figure 1).

### Correlations Between Graph Parameters and Clinical Variables

**Figure 4:**
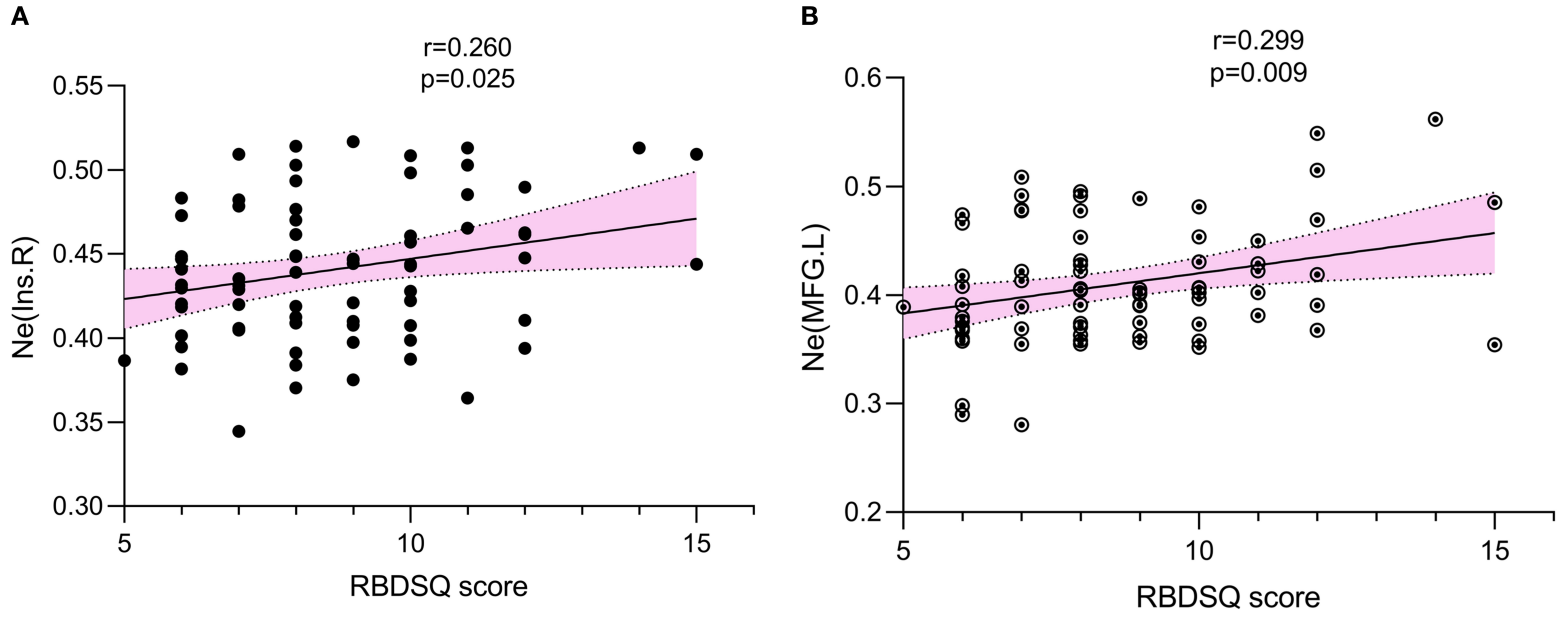
The association was explored by multiple linear regression. *p* < 0.05 was set significant. RBDSQ scores were positively associated with Ne of right insula (*r* = 0.260, *p* = 0.025) **(A)**, Ne of left middle frontal gyrus (*r* = 0.299, *p* = 0.009) **(B)**. Ne, nodal efficiency; RBDSQ, rapid eye movement sleep behavior disorder screening questionnaire.

Multiple linear regression analysis showed no significant correlation between RBDSQ scores and global parameters. However, RBDSQ scores were positively associated with the Ne of the right insula (*r* = 0.260, *p* = 0.025) and left middle frontal gyrus (*r* = 0.299, *p* = 0.009) (Figure 4).

## Discussion

In this study, DTI and graph theory methods were applied to evaluate the topological properties of WM networks in de novo and drug-naïve PD patients. All three groups presented similar small-world organization. Compared with PD-npRBD patients and HCs, PD-pRBD patients exhibited decreased Eg and increased Lp. Moreover, in nodal property analyses, the brain regions of the PD-pRBD group that had significantly altered Ne were widely distributed, mainly in neocortical and paralimbic regions compared with those in HCs. And, when compared with the PD-npRBD group, PD-pRBD group presented increased Ne in the right insula, left middle frontal gyrus, and decreased Ne in left temporal pole. In addition, a significant positive correlation between Ne in related brain regions and RBDSQ scores was detected in PD-pRBD patients.

In terms of global topological properties, the PD-pRBD group exhibited significantly decreased Eg and increased Lp values in WM structural networks compared with PD-npRBD patients and HCs. Eg and Lp values refer to the communication between distributed nodes and thus reflect the efficiency of global information transfer (Bullmore and Sporns, 2012). The decreased Eg and increased Lp values may indicate that the internodal organization of the whole brain was excessively damaged in PD-pRBD patients. Although the altered structural topological properties in PD with RBD have barely been explored, previous studies (Arnaldi et al., 2016) have indicated that PD with pRBD results in more severe motor and non-motor symptoms than PD without pRBD, suggesting more severe and widespread neurodegeneration. Thus, the normal topological network organization might be impacted; in other words, the separation and integration of brain networks might have been disrupted.

In this study, PD-pRBD patients showed extensive alterations in Ne when compared with HCs. The nodes were widely distributed in the neocortex and paralimbic system. A functional study showed an increase in the ALFF value of the prefrontal lobe in the PD-RBD group compared with the HC group (Li C. et al., 2017; Li D. et al., 2017). Another morphological study showed that the bilateral medial superior frontal lobe, orbitofrontal lobe and anterior cingulate cortex were thinner, and the gray matter volume in the frontal lobe, anterior cingulate gyrus and caudate nucleus were lower in the RBD patients compared to HC (Rahayel et al., 2018). These findings suggest that the pathophysiology of RBD in PD not only involves midbrain dysfunction, but also a wide range of cerebral cortex abnormalities, whose functional and structural abnormalities may be associated with motor and cognitive emotional dysfunction of PD-RBD. In addition, some other studies have shown that the paralimbic system is involved in the occurrence of RBD in PD. Kotagal et al. (2012) showed that compared with PD patients without RBD symptoms, subjects with RBD symptoms exhibited decreased cholinergic innervation in the paralimbic and thalamic cortices. Another MRI structure study of PD patients with RBD showed that the inferior temporal cortex, bilateral superior frontal gyri, and left rostral middle frontal cortex were extensively atrophied in PD patients compared to the controls, which suggests that the RBD symptoms of PD patients appeared along with significant paralimbic/limbic cortical changes (Pereira et al., 2019). The results of these studies were partially consistent with the findings of our study, which may indicate that the neocortex and paralimbic system play an important role in the development of PD with RBD.

When compared with the PD-npRBD group, altered Ne in PD-pRBD patients was distributed only in the right insula, left temporal pole and left middle frontal gyrus. Also, a positive correlation was discovered in our results between the Ne in right insula and left middle frontal gyrus with RBDSQ scores. The insular and temporal poles are considered part of the paralimbic system. The insula receives information from sensory pathways through the thalamus, integrates information about the state of the body, and sends output signals to other limbic-related structures, such as the amygdala and striatum (Furl, 2011). The temporal pole is associated with high-level cognitive processes: visual processing of complex objects, semantic processing in all modalities and socioemotional processing (Herlin and Navarro, 2021). The behaviors observed in RBD are violent, and the associated dreams are associated with feelings of sadness and fear. Thus, we speculated that the paralimbic cortex related to emotional control was involved in the pathogenesis of PD-RBD (Dauvilliers et al., 2018). Additionally, the middle frontal gyrus forms the frontal parietal network, known as a center of senior cognitive control center that regulates and integrates higher cognitive and emotional awareness (Kikinis et al., 2010). Studies have shown that PD patients with RBD perform poorly in cognitive tests (Boucetta et al., 2016; Gallea et al., 2017), suggesting that the abnormal increase of Ne in middle frontal gyrus may be related to cognitive regulation disorders in PD-pRBD. However, no difference in cognitive function was found among groups in our study, which may be because that patients with MoCa <23 were excluded from our study, although the number of such patients was very small (*n* = 4). In addition, the PD patients included in this study were still in the early stage of the disease, and had not yet shown obvious clinical manifestations of cognitive decline.A PET imaging study (Valli et al., 2021) suggested that extrastriatal dopamine regions including the prefrontal, insula, and limbic regions probably play a role in the progression of symptoms in PD individuals with RBD. In addition to dopaminergic denervation, notable deficits have been observed with the cholinergic pathway in the neocortical and limbic cortical regions of PD patients in a study (Kotagal et al., 2012). The exact pathological basis of RBD remains as yet undefined. A pathologic study reported that PD patients with RBD showed more severe a-synuclein pathology not only in brainstem but also in subcortical/limbic and cortical regions than those without RBD (Kalaitzakis et al., 2013).

Moreover, in hub analysis, the three groups of brain networks demonstrated 5 identical hub regions (3 association cortices; 2 subcortical): the left middle occipital gyrus, bilateral putamen and precuneus. Most of these regions have previously been reported as network hubs in the human brain (Tomasi and Volkow, 2011; van den Heuvel and Sporns, 2011, 2013). Compared with PD-npRBD group, bilateral temporal pole, left middle temporal gyrus lost their hub properties, while new hub regions were identified in left insula, right orbitofrontal gyrus, left precentral gyrus in PD-pRBD. Interestingly, we noticed that these hubs were also mainly located in the frontotemporal cortex and paralimbic regions. Hubs play an important role in maintaining network stability. This suggests that PD with RBD disrupts and reorganizes the hubs of the structural network, further proving that the neocortex and paralimbic regions may be involved in the neuropathological process of PD with RBD, which is consistent with the previous research results.

The study also had several limitations. First, we categorized PD patients according to RBDSQ scores rather than polysomnography results, which is the standard for diagnosing and quantitatively describing RBD. However, the RBDSQ score has high sensitivity and specificity for identifying RBD (Stiasny-Kolster et al., 2007). Second, this was a cross-sectional study of patients in the early stage of disease; future studies should include longitudinal follow-ups to further investigate the pathogenesis of PD-RBD. Third, we applied a deterministic fiber tracking method to estimate the structural connectivity between regions; this method could not determine the intersecting directions of multiple optical fibers. Therefore, to accurately measure the fiber trajectory, future studies should use a probabilistic tractography algorithm and diffusion spectral imaging technology. Fourth, other non-motor features that may affect brain network structure were not considered; future studies should include these features as control variables.

## Conclusion

This study indicated that the topological organization of the whole-brain structural connectome in PD-pRBD patients was impaired. Furthermore, we provide evidence that altered neocortical and paralimbic (mainly insula, temporal pole and middle frontal gyrus) nodal structural connections may play an important role in the pathophysiological mechanism underlying PD-RBD.

## Data Availability Statement

The original contributions presented in the study are included in the article/Supplementary Material, further inquiries can be directed to the corresponding author/s.

## Ethics Statement

The research was performed in accordance with the ethical standards laid down in the 1964 Declaration of Helsinki and its later amendments.

## Author Contributions

AC, YL, ZW, and XW had made a substantial contribution to the conception and drafting and revising the article. XR, XC, DL, and DC acquired the data. XH and JH had made a substantial contribution to the analysis and interpretation of the data. All of the authors gave final approval of the version to be published.

## Funding

This work was supported by the Natural Science Foundation of Guangdong Province, China (2021A1515011288), Science and Technology Program of Guangzhou (202102010020), and High-tech and Major Featured Project of Guangzhou (2019TS46).

## Conflict of Interest

The authors declare that the research was conducted in the absence of any commercial or financial relationships that could be construed as a potential conflict of interest.

## Publisher's Note

All claims expressed in this article are solely those of the authors and do not necessarily represent those of their affiliated organizations, or those of the publisher, the editors and the reviewers. Any product that may be evaluated in this article, or claim that may be made by its manufacturer, is not guaranteed or endorsed by the publisher.

## Supplementary Material

Supplementary Figure 1 The distribution of brain regions with significant differences in nodal efficiency in HC, PD-npRBD, and PD-pRBD groups. The size of the node reflects the value of the nodal efficiency. Different colors indicate a functional classification of brain regions: red: primary, purple: association, blue: paralimbic, green -limbic; yellow: subcortical. The abbreviations of the 90 brain regions are given in Supplementary Materials (Online Resource). L, left hemisphere; R, right hemisphere. Covariate, age, sex, years of education and GDS; GDS, Geriatric Depression Scale.

Supplementary Table 1 Cortical and subcortical regions of interest defined in the study.

Supplementary Table 2a Global properties of HC, PD-npRBD, and PD-pRBD patients.

Supplementary Table 2b Global properties of HC, PD-npRBD, and PD-pRBD patients.
