# Supplementary figures and images for: Disrupted Brain Structural Network Connection in de novo Parkinson's Disease With Rapid Eye Movement Sleep Behavior Disorder
Source: Front Hum Neurosci. 2022 Jul 19;16:902614. doi: 10.3389/fnhum.2022.902614 (PMC9344802; doi:10.3389/fnhum.2022.902614)

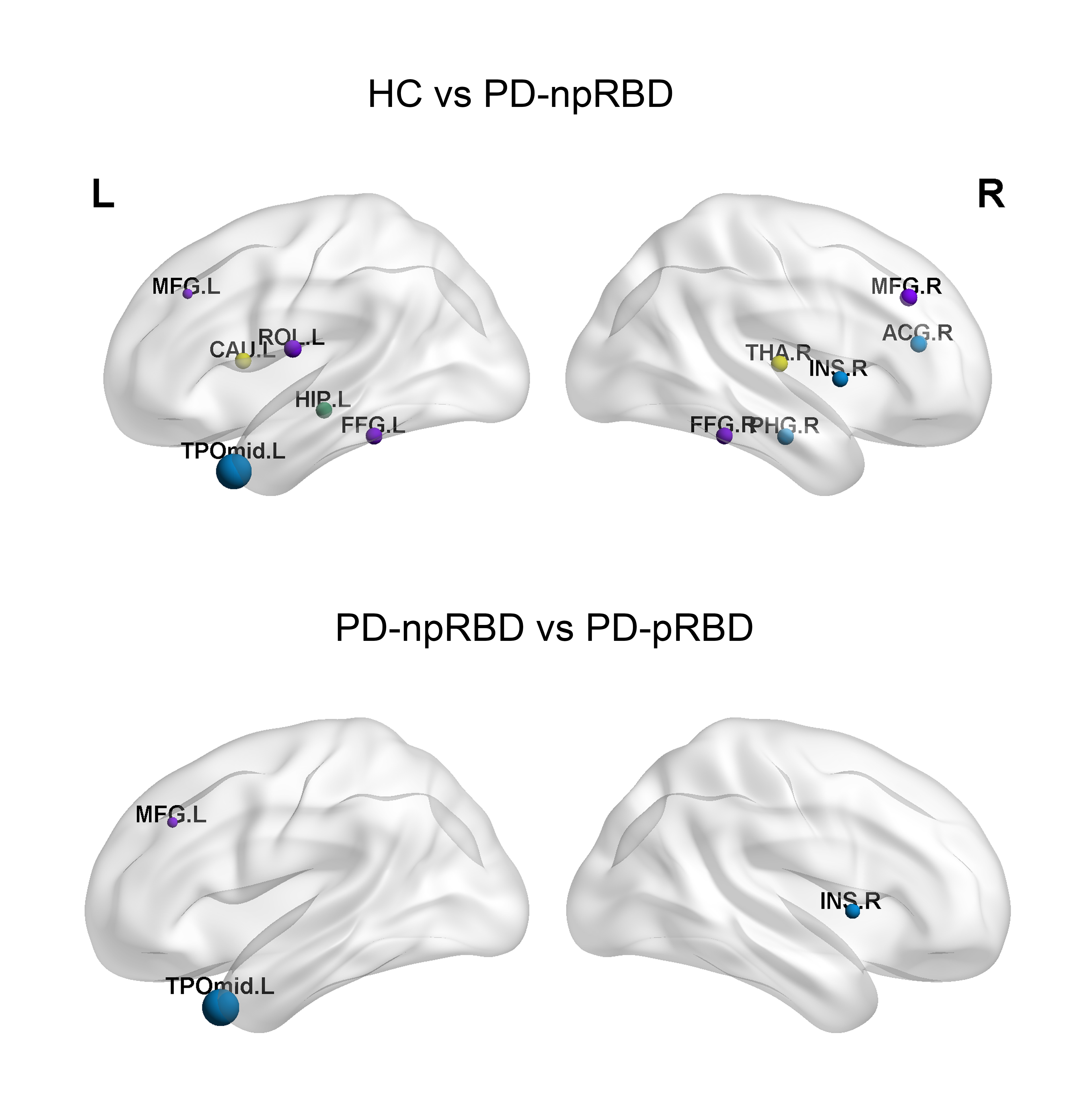

Supplement: Supplementary Figure 1 — The distribution of brain regions with significant differences in nodal efficiency in HC, PD-npRBD, and PD-pRBD groups. The size of the node reflects the value of the nodal efficiency. Different colors indicate a functional classification of brain regions: red: primary, purple: association, blue: paralimbic, green -limbic; yellow: subcortical. The abbreviations of the 90 brain regions are given in Supplementary Materials (Online Resource). L, left hemisphere; R, right hemisphere. Covariate, age, sex, years of education and GDS; GDS, Geriatric Depression Scale. [file Image_1.TIF]
