# Supplementary material for: Disrupted Brain Structural Network Connection in de novo Parkinson's Disease With Rapid Eye Movement Sleep Behavior Disorder
Source: Front Hum Neurosci. 2022 Jul 19;16:902614. doi: 10.3389/fnhum.2022.902614 (PMC9344802; doi:10.3389/fnhum.2022.902614)
Supplement: Supplementary Table 1 — Cortical and subcortical regions of interest defined in the study. [file Table_1.DOCX]

supplementary table 1: Cortical and subcortical regions of interest defined in the study

| Index | Region | Abbr. | Index | Region | Abbr. |
| --- | --- | --- | --- | --- | --- |
| 1,2 | Precental gyrus | PreCG | 47,48 | Lingual gyrus | LING |
| 3,4 | Superior frontal gyrus, dorsolateral | SFGdor | 49,50 | Superior occipital gyrus | SOG |
| 5,6 | Superior frontal gyrus, orbital part | ORBsup | 51,52 | Middle occipital gyrus | MOG |
| 7,8 | Middle frontal gyrus | MFG | 53,54 | Inferior occipital gyrus | IOG |
| 9,10 | Middle frontal gyrus, orbital part | ORBmid | 55,56 | Fusiform gyrus | FFG |
| 11,12 | Inferior frontal gyrus, opercular part | IFGoperc | 57,58 | Postcentral gyrus | PoCG |
| 13,14 | Inferior frontal gyrus, triangular part | IFGtriang | 59,60 | Superior parietal gyrus | SPG |
| 15,16 | Inferior frontal gyrus, orbital part | ORBinf | 61,62 | Inferior parietal, but supramarginal and angular gyri | IPL |
| 17,18 | Rolandic operculum | ROL | 63,64 | Supramarginal gyrus | SMG |
| 19,20 | Supplementary motor area | SMA | 65,66 | Angular gyrus | ANG |
| 21,22 | Olfactory cortex | OLF | 67,68 | Precuneus | PCUN |
| 23,24 | Superior frontal gyrus, medial | SFGmed | 69,70 | Paracentral lobule | PCL |
| 25,26 | Superior frontal gyrus, medial orbital | ORBsupmed | 71,72 | Caudate nucleus | CAU |
| 27,28 | Gyrus rectus | REC | 73,74 | Lenticular nucleus, putamen | PUT |
| 29,30 | Insula | INS | 75,76 | Lenticular nucleus, pallidum | PAL |
| 31,32 | Anterior cingulate and paracingulate gyri | ACG | 77,78 | Thalamus | THA |
| 33,34 | Median cingulate and paracingulate gyri | DCG | 79,80 | Heschl gyrus | HES |
| 35,36 | Posterior cingulate gyrus | PCG | 81,82 | Superior temporal gyrus | STG |
| 37,38 | Hippocampus | HIP | 83,84 | Temporal pole: superior temporal gyrus | TPOsup |
| 39,40 | Parahippocampal gyrus | PHG | 85,86 | Middle temporal gyrus | MTG |
| 41,42 | Amygdala | AMYG | 87,88 | Temporal pole: middle temporal gyrus | TPOmid |
| 43,44 | Calcarine fissure and surrounding cortex | CAL | 89,90 | Inferior temporal gyrus | ITG |
| 45,46 | Cuneus | CUN |  |  |  |

The regions are listed according to a prior template obtained from an AAL atlas; odd number represents the corresponding brain regions in left hemisphere; even number indicates the specific brain regions in right hemisphere.
