# Supplementary material for: Disrupted Brain Structural Network Connection in de novo Parkinson's Disease With Rapid Eye Movement Sleep Behavior Disorder
Source: Front Hum Neurosci. 2022 Jul 19;16:902614. doi: 10.3389/fnhum.2022.902614 (PMC9344802; doi:10.3389/fnhum.2022.902614)
Supplement: Supplementary Table 2a — Global properties of HC, PD-npRBD, and PD-pRBD patients. [file Table_2.DOCX]

**Supplementary TABLE 2a.** Global properties of HC, PD-npRBD and PD-pRBD patients

|  | | HC(n=59) | PD-npRBD  (n=97) | | PD-pRBD  (n=56) | *P* value | | Post hoc analysis *P* value | | | | |
| --- | --- | --- | --- | --- | --- | --- | --- | --- | --- | --- | --- | --- |
|  |  |  |  |  |  |  |  | HC vs PD-npRBD | HC vs PD-pRBD | | PD-npRBD vs PD-pRBD | |
| Eg | 0.435 0.456 | | | 0.429 | | | 0.004* 0.091 | | | 0.002* 0.005* | |  |
| Lp | 2.382 2.373 | | | 2.439 | | | 0.006* 0.084 | | | 0.006* 0.004* | |  |
| Eloc | 0.725 0.724 | | | 0.725 | | | 0.182 / | | | / / | |  |
| Cp | 0.519 0.510 | | | 0.515 | | | 0.293 / | | | / / | |  |
| σ | 3.459 3.380 | | | 3.472 | | | 0.171 / | | | / / | |  |
| γ | 4.516 4.524 | | | 4,519 | | | 0.253 / | | | / / | |  |
| λ | 1.190 1.191 | | | 1.190 | | | 0.364 / | | | / / | |  |
|  | |  |  | |  |  | |  |  | |  | |

**Covariate:** age, sex, and years of education

**Supplementary TABLE 2b.** Global properties of HC, PD-npRBD and PD-pRBD patients

|  | | HC(n=59) | PD-npRBD  (n=97) | | PD-pRBD  (n=56) | *P* value | | Post hoc analysis *P* value | | | | |
| --- | --- | --- | --- | --- | --- | --- | --- | --- | --- | --- | --- | --- |
|  |  |  |  |  |  |  |  | HC vs PD-npRBD | HC vs PD-pRBD | | PD-npRBD vs PD-pRBD | |
| Eg | 0.435 0.456 | | | 0.429 | | | 0.004* 0.075 | | | 0.002* 0.006* | |  |
| Lp | 2.382 2.373 | | | 2.439 | | | 0.005* 0.089 | | | 0.005* 0.003* | |  |
| Eloc | 0.725 0.724 | | | 0.725 | | | 0.182 / | | | / / | |  |
| Cp | 0.519 0.510 | | | 0.515 | | | 0.293 / | | | / / | |  |
| σ | 3.459 3.380 | | | 3.472 | | | 0.171 / | | | / / | |  |
| γ | 4.516 4.524 | | | 4,519 | | | 0.253 / | | | / / | |  |
| λ | 1.190 1.191 | | | 1.190 | | | 0.364 / | | | / / | |  |
|  | |  |  | |  |  | |  |  | |  | |

**Covariate:** age, sex, years of education and GDS (GDS: Geriatric Depression Scale)

HC: healthy control group, PD-npRBD: Parkinson’s disease with no probably REM Sleep Behavior Disorder, PD-pRBD: Parkinson’s disease with probably REM Sleep Behavior Disorder; Eg: global efficiency, Lp: shortest path length, Eloc: local efficiency, Cp: cluster coefficiency, σ, γ, λ: the “small worldness” attribute. F: one-way ANOVA test, t: two-tailed t test,Significant values were adjusted by the Bonferroni correction for multiple tests. *The significance threshold was set at p<0.05.
